# Immediate vs. culture-initiated antibiotic therapy in suspected non-severe ventilator-associated pneumonia: a before–after study (DELAVAP)

**DOI:** 10.1186/s13613-024-01243-z

**Published:** 2024-02-27

**Authors:** Maëlle Martin, Solène Forveille, Jean-Baptiste Lascarrou, Amélie Seguin, Emmanuel Canet, Jérémie Lemarié, Maïté Agbakou, Luc Desmedt, Gauthier Blonz, Olivier Zambon, Stéphane Corvec, Aurélie Le Thuaut, Jean Reignier

**Affiliations:** 1 Nantes Université, CHU Nantes, Médecine Intensive Réanimation, Nantes, France; 2 Nantes Université, CHU Nantes, Institut de Biologie des Hôpitaux de Nantes, Service de Bactériologie Et Des Contrôles Microbiologiques, Nantes, France; 3 Nantes Université, CHU Nantes, Plateforme de méthodologie et biostatistique, Direction de la recherche et de l’innovation, Nantes, France; 4 Nantes Université, CHU Nantes, Médecine Intensive Réanimation, Movement - Interactions - Performance, MIP, UR 4334 Nantes, France

**Keywords:** Critical care, Ventilator-associated pneumonia, Antibiotic therapy, Antibiotic stewardship

## Abstract

**Background:**

Ventilator-associated pneumonia (VAP) is the leading nosocomial infection in critical care and is associated with adverse outcomes. When VAP is suspected, starting antibiotic therapy (AT) immediately after pulmonary sampling may expose uninfected patients to unnecessary treatment, whereas waiting for bacteriological confirmation may delay AT in infected patients. As no robust data exist to choose between these strategies, the decision must balance the pre-test diagnostic probability, clinical severity, and risk of antimicrobial resistance. The objective of this study in patients with suspected non-severe VAP was to compare immediate AT started after sampling to conservative AT upon receipt of positive microbiological results. The outcomes were antibiotic sparing, AT suitability, and patient outcomes.

**Methods:**

This single-center, before–after study included consecutive patients who underwent distal respiratory sampling for a first suspected non-severe VAP episode (no shock requiring vasopressor therapy or severe acute respiratory distress syndrome). AT was started immediately after sampling in 2019 and upon culture positivity in 2022 (conservative strategy). The primary outcome was the number of days alive without AT by day 28. The secondary outcomes were mechanical ventilation duration, day-28 mortality, and AT suitability (active necessary AT or spared AT).

**Results:**

The immediate and conservative strategies were applied in 44 and 43 patients, respectively. Conservative and immediate AT were associated with similar days alive without AT (median [interquartile range], 18.0 [0–21.0] vs. 16.0 [0–20.0], *p* = 0.50) and without broad-spectrum AT (*p* = 0.53) by day 28. AT was more often suitable in the conservative group (88.4% vs. 63.6%, *p* = 0.01), in which 27.9% of patients received no AT at all. No significant differences were found for mechanical ventilation duration (median [95%CI], 9.0 [6–19] vs. 9.0 [6–24] days, *p* = 0.65) or day-28 mortality (hazard ratio [95%CI], 0.85 [0.4–2.0], *p* = 0.71).

**Conclusion:**

In patients with suspected non-severe VAP, waiting for microbiological confirmation was not associated with antibiotic sparing, compared to immediate AT. This result may be ascribable to low statistical power. AT suitability was better with the conservative strategy. None of the safety outcomes differed between groups. These findings would seem to allow a large, randomized trial comparing immediate and conservative AT strategies.

**Supplementary Information:**

The online version contains supplementary material available at 10.1186/s13613-024-01243-z.

## Background

Ventilator-associated pneumonia (VAP) is the leading nosocomial infection in intensive care units (ICUs) [1]. VAP is associated with longer invasive mechanical ventilation (iMV), increased antibiotic consumption, and higher hospital costs [2]. Whether VAP is associated with higher mortality remains controversial [3].

Identifying VAP is challenging, as no specific diagnostic criteria exist for ICU patients [4]. Even when a combination of clinical, laboratory, and radiological findings indicate a high pre-test probability of VAP, the distal respiratory sample cultures confirm the suspicion in only about half the cases [5]. Consequently, AT given when VAP is suspected, before the culture results are available, unnecessarily exposes many patients to the side effects of antimicrobials. Moreover, unnecessary AT adversely affects public health by increasing the risk of selecting resistant bacteria [6–8]. In a before–after, propensity-score-matched study, culture-initiated AT, started only after microbiological confirmation of infection, was associated with fewer ICU-acquired extended-spectrum beta-lactamase-producing (ESBL) *Enterobacterales* infections [9].

Whether the conservative strategy of culture-initiated AT carries risks to patients is unclear. In the above-cited study [9], in which this conservative strategy was reserved for patients without severity criteria, all-cause ICU mortality was significantly lower than with the immediate strategy of starting empirical AT immediately after microbiological sample collection. Another before–after study in surgical patients with any type of ICU-acquired infection demonstrated that the conservative strategy was associated with significantly lower values for all-cause mortality, inappropriate initial AT, and mean AT duration [10]. The results from these two observational studies should be interpreted with caution despite the adjustment of the analyses, since the development of severity criteria may have led to rescue AT before culture-result availability in the conservative-strategy groups. We are not aware of any randomized controlled trials comparing the conservative and immediate strategies.

The objective of this before–after ICU study was to further compare the immediate and conservative AT strategies for managing VAP in patients without severity criteria. The primary outcome was days alive without AT by day 28. The secondary outcomes included day-28 mortality, iMV duration, and AT suitability.

## Methods

The study was approved by the ethics committee of the French Intensive Care Society (*Société de Réanimation de Langue Française*) on December 24, 2021 (CE SRLF 21-110). The need for informed consent was waived because both strategies were part of standard ICU practice, in the absence of published data supporting one over the other, and because both were implemented according to protocols routinely applied in the study ICU during two different time periods. The study was registered on ClinicalTrials.gov (NCT05205525).

## Design

Consecutive patients aged 18 years or older who received iMV for longer than 48 h in the medical ICU of the Nantes University Hospital (Nantes, France) and underwent distal respiratory sampling for a suspected, first VAP episode were screened for eligibility throughout 2019 and 2022. Screening was retrospective in 2019 and prospective in 2022. One-year periods were studied to avoid bias from seasonal factors. We did not study 2020 or 2021, during which nearly all patients given iMV had acute respiratory distress syndrome (ARDS) due to COVID-19.

We did not include patients with severity criteria around the time of VAP suspicion. No validated definition of severe VAP has been reported. Our choice of severity criteria was based on documented associations linking severe ARDS and septic shock to high mortality [11, 12] and suggesting poorer VAP outcomes in patients with low PaO_2_/FiO_2_ or cardiovascular failure [13–15]. The severity criteria leading to non-inclusion in our study were, thus, shock requiring vasopressor therapy or the ARDS with the onset or severe worsening of hypoxemia (partial pressure of oxygen in arterial blood/fraction of inspired oxygen [PaO_2_/FiO_2_] < 150 with 60% FiO_2_ and 10 cmH_2_O peak expiratory pressure, or veno-venous extracorporeal membrane oxygenation). A 2018 analysis of a large prospective multinational database study of VAP demonstrated significantly higher mortality among patients with vs. without immunosuppression [16]. We consequently also did not include patients with immunosuppression defined as neutropenia < 1 G/L or immunosuppressive treatment (including corticosteroid therapy for longer than 1 month or in a dosage > 0.5 mg/kg/day). The other non-inclusion criteria were ongoing AT with a predicted duration ≥ 4 weeks (e.g., for endocarditis, spondylodiscitis, or an abscess), previous episode of suspected VAP with sampling and/or AT, and previous inclusion in the study. Patients on AT of predicted duration below 4 weeks were included.

The 25-bed medical ICU at the Nantes University Hospital has five board-certified intensivists who apply written ICU protocols, notably for ICU-acquired infections, sedation, and iMV weaning. The ICU team includes staff members specifically trained in infectious diseases and AT. The ICU belongs to the nationwide network REA-REZO [17], which monitors and assesses the risk of healthcare-associated infections and antibiotic resistance in adult critical care and collaborates with the French antibiotic-use surveillance system SPARES.

## Definitions

### Suspected ventilator-associated pneumonia

VAP is defined as pneumonia with onset between 48 h after intubation and 48 h after extubation [18]. As recommended, all patients on iMV for longer than 48 h underwent a physical examination and laboratory tests at least once a day to look for signs suggesting VAP. Chest radiographs were obtained when deemed necessary. As indicated in the 2018 recommendations by French critical-care societies [19], VAP was suspected in patients having:

- At least three of the following clinical criteria: body temperature ≥ 38.5 °C or ≤ 35.5 °C, blood leucocyte count > 12 000/mL or < 4000/mL, purulent tracheobronchial aspirate, increased oxygen requirements (persistent need for an FiO2 at least 20% higher than the mean value during the last 48 h)
- And then, a new and/or changing chest-radiograph infiltrates.

Patients with suspected VAP underwent distal protected respiratory sampling by either bronchoalveolar lavage (BAL) or telescoping plugged catheter (TPC), as deemed best by the intensivist in charge [20].

### Confirmed ventilator-associated pneumonia

Confirmed VAP was defined as BAL or TPC cultures showing bacterial counts above ≥ 10^4^ CFU/mL or ≥ 10^3^ CFU/mL, respectively, and/or a positive blood culture with no identified source of infection other than VAP.

### Antibiotic therapy suitability

We recorded whether the antibiotics administered were effective against the recovered microorganisms (active/inactive AT); the use of AT in patients with negative culture results (unnecessary AT); and the absence of AT in patients with negative cultures (spared AT). Active AT was defined as a regimen active on all the microorganism(s) recovered in concentrations above pre-specified thresholds, as shown by antibiotic susceptibility testing (AST). AT that did not meet this criterion was defined as inactive. Unnecessary AT was AT given to a patient subsequently found to have negative cultures. Spared AT was absence of AT administration to a patient whose cultures were negative. Suitable AT was defined as active or spared AT and unsuitable AT as inactive or unnecessary AT.

In the group managed using the conservative strategy, we recorded the number of patients given culture-initiated AT (AT upon receipt of positive culture results) and the number given rescue AT (AT before receipt of the culture results due to the development of shock, worsening severe hypoxemia, and/or a positive blood culture).

## Immediate and conservative antibiotic therapy strategies

National and international guidelines issued in 2016 and 2017, which constitute the most recent versions, have been applied continuously in our ICU since 2017, with the adjustments appropriate for our local ecology. No change occurred between 2019 and 2022.

Patients with suspected VAP immediately underwent distal respiratory sampling and were then assessed for severity criteria precluding study inclusion (shock, worsening hypoxemia, immunosuppression, ongoing AT with an expected duration ≥ 4 weeks). Patients with severity criteria were immediately given empirical AT active against the bacteria usually responsible for VAP in our ICU. These patients were not included in the present study, whatever the period.

**Fig. 1:**
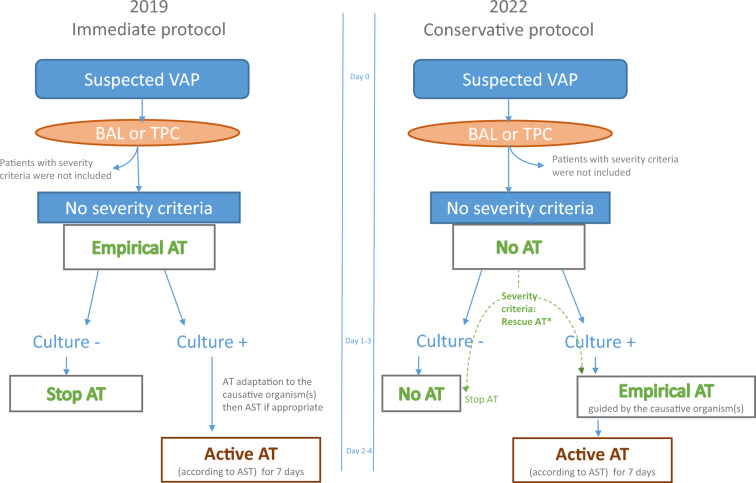
Protocol for respiratory sampling and antibiotic therapy. AT: antibiotic therapy, VAP: ventilator-associated pneumonia; BAL: bronchoalveolar lavage; TPC: telescoping plugged catheter; -: negative; + : positive; AST: antibiotic susceptibility testing. *Rescue AT: empirical AT given before the culture results became available, in a patient in the conservative group, due to the development of shock or worsening severe hypoxemia and/or to a positive blood culture after sampling

The immediate AT strategy was used throughout 2019. Immediately after distal respiratory sampling, patients with suspected VAP received empirical AT active against the bacteria usually responsible for VAP in our ICU. When selecting the antibiotic regimen, risk factors for antimicrobial resistance in the individual patient, previous iMV duration, and the current local ecology were considered [21] (Fig. 1). Once the culture results became available, the antibiotic regimen was changed if found to be inactive and de-escalated if allowed by the recovered microorganisms and the AST results. The total duration of active AT was 7 days.

The conservative AT strategy was applied throughout 2022. Patients with suspected VAP immediately underwent distal respiratory sampling, but were given AT only if severity criteria and/or a positive blood culture developed before the culture results became available (rescue AT) or if the culture results were positive. Gram stain was not used to guide AT because it was not available 24 h a day and because poor concordance between Gram stain results and final cultures has been reported [22]. The antimicrobials were selected based on the identified cultured microorganisms then adjusted according to the AST findings. Again, the total duration of active AT was 7 days (Fig. 1). No other procedures that may have affected the management of suspected VAP (AT type, staffing level, devices) changed during the study period.

## Data collection and outcome measures

Data for each patient were collected in an electronic health record (Millenium, Cerner, Kansas City, MO in 2019 and ICCA, Philips, Amsterdam, The Netherlands in 2022) then transferred to an electronic case report form (Wepi, Epiconcept, Paris, France).

The primary outcome was the number of days alive without AT by day 28. AT days were the number of calendar days with AT, irrespective of the number of antimicrobial agents and number of doses given. Patients who died before day 28 while still on AT were classified as having 0 days alive without AT.

Secondary outcomes included the number of days alive by day 28 without broad-spectrum AT (ceftazidime, piperacillin/tazobactam, cefepime, fluoroquinolones, carbapenems, or new antimicrobials for multidrug-resistant Gram-negative bacilli [23]), number of days alive without carbapenem by day 28, AT suitability, and AT safety (in-ICU *Clostridium difficile* infections and antibiotic allergies). Other secondary outcomes were iMV duration; ventilator-free days; ICU stay length; day-28 mortality; incidences of VAP-related complications (abscess and bacteremia); subsequent VAP; and in-ICU infections by multiresistant bacteria (MRB), The MRBs were methicillin-resistant *Staphylococcus aureus* (MRSA), glycopeptide-intermediate *S. aureus* (GISA), vancomycin-resistant *Enterococcus* (VRE), ESBL *Enterobacterales*, carbapenemase-producing *Enterobacterales* (CPE), and imipenem-resistant *Acinetobacter baumannii* (IRAB).

## Statistical analyses

The study variables were described as median [interquartile range]. The numbers of days alive without AT, without broad-spectrum AT, and without carbapenem by day 28 were compared between groups by applying the Wilcoxon signed-rank test. Comparisons of the incidences of in-ICU MRB infections, in-ICU *C. difficile* infections, antibiotic allergies, and VAP-related abscess and bacteremia were with Fisher’s exact test. ICU stay length and iMV duration were compared by Fine-and-Gray survival analysis with death as a competing risk. For the comparison of day-28 mortality, we built a Cox proportional hazards model. The other variables were compared using the Wilcoxon signed-rank test if continuous and the chi-square or Fisher’s exact test if categorical. Adverse events were further compared between the two strategies specifically in those patients with confirmed VAP. Finally, we performed linear regression analyses adjusted on PaO_2_/FiO_2_ at VAP suspicion, respiratory reason for iMV, and time from intubation to VAP suspicion.

The statistical analyses were performed using SAS software, version 9.4 (SAS Institute, Cary, NC). Values of *p* lower than 0.05 were taken to indicate significant differences.

## Results

### Patients

**Fig. 2:**
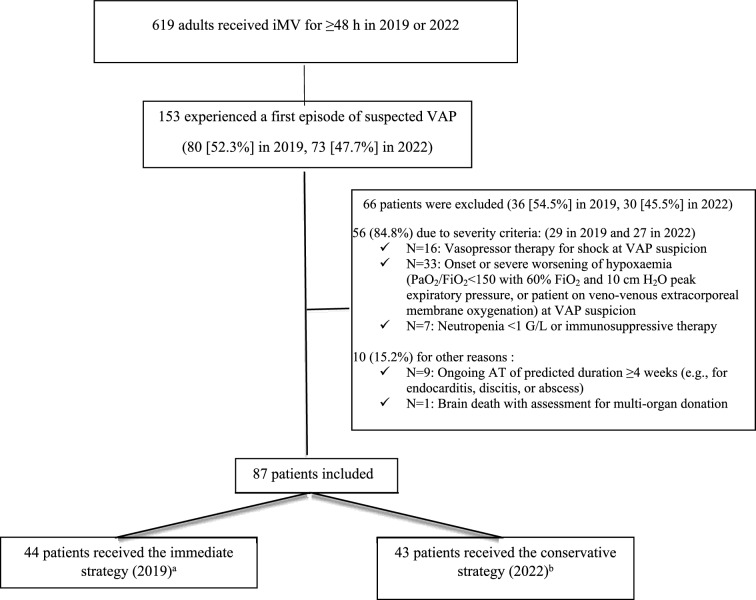
Patient flow chart. ICU: intensive care unit; iMV: invasive mechanical ventilation; VAP: ventilator-associated pneumonia. **a** Deviations from the immediate strategy occurred in 3 of these 44 patients, for the following reasons: 1 patient had suspected early VAP and, at sampling, was already on AT for non-VAP-related reasons, with a regimen deemed appropriate as empirical VAP therapy; 1 patient developed signs consistent with suspected VAP during a window in antibiotic therapy given for complex peritonitis reason why AT was delayed; and 1 patient with neuromuscular disease had radiographic features suggesting atelectasis in part of a lung infiltrate, that made AT delayed for low likelihood of infection. **b** Deviations from the conservative strategy occurred in 2 of these 43 patients, because they were managed by an intensivist who was new to the ICU and not yet fully aware of the study protocol. In both patients, the deviation consisted in starting antibiotics immediately after lung sampling. Overall protocol compliance was thus 82/87 (94.3%)

Figure 2 is the patient flow chart. Table 1 shows the baseline characteristics of the study patients were evenly balanced between the two groups. VAP was confirmed in 56/87 (64.5%) patients overall, 30/44 (68.2%) patients in the immediate group, and 26/43 (60.5%) patients in the conservative group (*p* = 0.45). Median time from intubation to VAP confirmation was 6.0 [3.0–8.0] and 7.0 [5.0–10.0] days in the immediate and conservative groups, respectively. Median time from VAP suspicion and sampling to culture-result availability was 41.8 [23.7–60.2] hours. Additional file 1: Table S1 lists the pathogens recovered from the distal respiratory samples. The most common were *Enterobacterales* (58.2%, of which 21.8% were group III *Enterobacterales* and 20% were *Escherichia coli*) and *S. aureus* (40%). *Pseudomonas aeruginosa* was identified in 7.3% of patients with confirmed VAP. No MRBs were acquired during the ICU stay.

**Table 1:** Main baseline characteristics of the 87 study patients

	Immediate n = 44	Conservative n = 43	*p* value
Age, y, median [IQR]	55.3 [49.9–65.1]	59.3 [52.3–69.3]	0.32
Females, n (%)	14 (31.8)	17 (39.5)	0.45
Immunodeficiency, n (%)	0 (0)	3 (6.9)^a^	0.12
AT within 48 h of ICU admission, n (%)	30 (68.2)	27 (62.8)	0.60
SAPS II, median [IQR]	55.0 [42.5–64.0]	58.0 [42.0–68.0]	0.75
Knaus Chronic Health Status Score [24], n (%)			
A	12 (27.3)	10 (23.3)	0.59
B	22 (50.0)	26 (60.5)	
C	10 (22.7)	7 (16.3)	
Reason for iMV, n (%)			
Neurologic	28 (63.6)	18 (41.9)	0.10
Respiratory	15 (34.1)	23 (53.49)	
Other	1 (2.27)	2 (4.65)	
COVID-19 pneumonia	0 (0)	9 (20.9%)	0.001
Time from intubation to VAP suspicion, days, median [IQR]	6.0 [3.0–8.0]	7.0 [5.0–10.0]	0.10
Patients with confirmed VAP	30 (68.2%)	26 (60.5%)	0.45
20% FiO_2_ increase around VAP suspicion, n (%)	34 (77.3)	33 (76.7)	0.95
Lowest PaO_2_/FiO_2_ around VAP suspicion, median [IQR]	172 [161–197]	172 [153–233]	0.94
Lowest/Highest body temperature around VAP suspicion, median [IQR]	38.65 [38.1–39.2]	38.5 [38.1–38.9]	0.14
Lowest/Highest leucocyte count around VAP suspicion, median [IQR]	14.3 [10.3–18.5]	11.9 [8.5–18.9]	0.32
Purulent respiratory secretions at VAP suspicion, n (%)	32 (72.7)	36 (83.7)	0.21

AT: antibiotic therapy; ICU: intensive care unit; SAPS II: Simplified Acute Physiology Score version II; iMV: invasive mechanical ventilation; VAP: ventilator-associated pneumonia

^a^These three patients were taking biological agents deemed by the investigators to carry no increased risk of VAP or severe VAP

### Antibiotic sparing and suitability

Table 2 shows that no significant between-group differences were found for the number of days alive without AT by day 28 (primary outcome) or for the numbers of days alive without broad-spectrum AT or without carbapenem by day 28.

**Table 2:** Antibiotic use and other outcome measures

	Immediate n = 44	Conservative n = 43	*p* value
Days alive without AT by day 28, median [IQR]	16.0 [0.0–20.0]	18.0 [0.0–21.0]	0.50
Days alive without broad-spectrum AT by day 28, median [IQR]	23.5 [5.0–26.0]	25.0 [0.0–28.0]	0.53
Days alive without carbapenem by day 28, median [IQR]	28.0 [9.0–28.0]	28.0 [0.0–28.0]	0.65
iMV duration, days, median [95% confidence interval] HR [95%CI]	9.0 [6.0–24.0] 1.1 [0.7–1.8]	9.0 [6.0–19.0]	0.65
Ventilator-free days, median [IQR]	18.5 [0.0–23.0]	16.0 [0.0–22.0]	0.99
ICU stay, days, median [95% confidence interval]	9.0 [6.0–14.0]	13.0 [8.0–17.0]	0.71
Day-28 mortality, n (%) HR [95%CI]	11 (25.0) 0.8 [0.4–2.0]	11 (25.6)	0.71
VAP-related abscess, n (%)	0	2 (4.6)	0.24
VAP-related bacteraemia, n (%)	0	2 (4.6)	0.24
Subsequent VAP, n (%)			
Recurrence^a^	3 (6.8)	2 (4.6)	0.11
Superinfection^a^	2 (4.5)	0 (0)	
First VAP after suspected unconfirmed VAP	0 (0)	4 (9.3)	
MRB^b^, n (%)	0	0	-
*Clostridium difficile* infections, n (%)	0	0	-
Antibiotic allergy, n (%)	2 (4.5)	0	0.49

ICU: intensive care unit; iMV: endotracheal mechanical ventilation; HR: hazard ratio; 95%CI: 95% confidence interval; VAP: ventilator-associated pneumonia

^a^Recurrence was defined as improvement in the manifestations (fever, secretions, vasopressor needs, inflammatory biomarkers, and chest radiograph infiltrates) after 7-day treatment with at least one antibiotic active on all documented bacteria, followed by the return of these manifestations and growth in new distal respiratory specimens of at least one bacterial species in significant concentrations

The same scenario with growth of at least one of the initial causative bacteria defined relapse (n = 0); otherwise, it was considered to be a superinfection [25]

^b^MRB indicates any of the following multiresistant bacteria: methicillin-resistant *Staphylococcus aureus* (MRSA), glycopeptide-intermediate *Staphylococcus aureus* (GISA), vancomycin-resistant *Enterococcus* (VRE), extended-spectrum beta-lactamase-producing *Enterobacterales* (ESBL-e), carbapenemase-producing *Enterobacterales* (CPE), and imipenem-resistant *Acinetobacter baumannii* (IRAB)

Median time from respiratory sampling to AT was 0.5 [0.1–1.5] hours with the immediate strategy and 23.8 [14.4–39.8] hours with the conservative strategy. Empirical AT was defined as AT started before the receipt of AST results, either before or upon the receipt of culture results. Thus, the only patients not given empirical AT were the 12 conservative-group patients whose culture results were negative, who did not receive rescue AT, and in whom the protocol was followed. The most widely used empirical medications were piperacillin/tazobactam for immediate AT (54.5%) and amoxicillin-clavulanic acid for conservative AT (38.7%) (Additional file 1: Table S2). Empirical dual therapy was used in 4.5% and 19.3% patients in the immediate and conservative groups, respectively (*p* = 0.15). The proportion of patients with AT de-escalation (defined as empirical AT stopped or number of empirical antimicrobials decreased or spectrum of empirical AT narrowed, based on AST results) was 63.6% (28/44) with the immediate strategy vs. 45.2% (14/31) with the conservative strategy (*p* = 0.11). Finally, in the conservative group, 12/43 (27.9%) patients were spared AT (negative cultures).

Empirical AT was significantly more often suitable (active or spared) with the conservative strategy than with the immediate strategy (84.4% vs. 63.6%, *p* = 0.01) (Table 3). Of the 43 patients in the conservative group, 19 (44.2%) received AT because their cultures were positive, 10 (23.3%) received rescue AT due to the development of severity criteria (n = 8) and/or a positive blood culture (n = 2) while waiting for the culture results, and 2 received AT before the culture results because the protocol was not followed. Thus, 31 patients in the conservative group received empirical AT. The cultures were negative in 3 patients given rescue AT. In the remaining 7 patients, the rescue regimen was active. The cultures were negative in both patients for whom the protocol was not followed. Thus, unnecessary AT was given to 5/43 (11.6%) patients in the conservative group, compared to 14/44 (31.8%) in the immediate group.

**Table 3:** Antibiotic therapy (AT) suitability

	Immediate n = 44	Conservative n = 43	*p* value
Active AT^a^	28 (63.6%)	26 (60.5%)	0.76
Inactive AT	2 (4.5%)	0 (0.0%)	0.49
Unnecessary AT^b^	14 (31.8%)	5 (11.6%)^b^	0.02
Spared AT^c^	-	12 (27.9%)	-
Suitable AT^d^	28 (63.6%)	38 (88.4%)	0.01
Culture-initiated AT^e^	3 (6.8%)^e^	19 (44.2%)	0.001
Rescue AT^f^	-	10 (23.3%)	-

^a^Active AT: at least one antimicrobial agent active (based on antibiotic susceptibility testing) against each microorganism cultured from a distal respiratory sample in a concentration above the pre-specified threshold (otherwise, inactive AT)

^b^Unnecessary AT: AT given then negative culture results (immediate group or rescue AT in the conservative group plus 2 patients in the conservative group managed in 2022 but in whom the protocol for that year was not followed)

^c^Spared AT: no AT given and negative culture results (conservative group only)

^d^Suitable AT: active AT or spared AT (both groups)

^e^Culture-initiated AT: AT given only when the culture results became available (conservative group and 3 patients in the immediate group managed in 2019 but in whom the protocol for that year was not followed)

^f^Rescue AT: AT given before the culture results became available in the conservative group, due to the development of shock, worsening severe hypoxemia and/or to a positive blood culture after sampling

### Safety

Table 2 shows that no significant between-group differences were demonstrated for iMV duration, ventilator-free days, ICU stay length, day-28 mortality, VAP-related bacteremia or abscess, subsequent VAP, MRB infections, *C. difficile* infection, or allergies.

Similarly, no significant differences were observed for these variables in the analysis confined to patients with microbiologically documented VAP (Additional file 1: Table S3) or in the analyses adjusted on PaO_2_/FiO_2_ at VAP suspicion, respiratory reason for iMV, and time from intubation to VAP suspicion (Additional file 1: Table S4).

## Discussion

In this single-center before–after study, a conservative strategy of initiating AT only upon microbiological confirmation of VAP was not associated with fewer days alive without AT by day 28, compared to an immediate strategy of starting AT when VAP was suspected. Moreover, no significant differences were evidenced between the two strategies for days alive without broad-spectrum AT or carbapenem by day 28, iMV duration, ICU stay length, or day-28 mortality. Over a quarter of the patients in the conservative group were spared unnecessary AT.

The number of days alive without AT by day 28 (primary outcome) is not significantly greater in the conservative group may be ascribable to insufficient statistical power due to the limited number of patients. Another small study, with 186 patients managed for suspected non-severe VAP, also showed no difference in days on AT between the immediate and conservative strategies [5]. Patient outcomes were similar in our two groups, and a fifth of patients in the conservative group were spared AT. In contrast, in two larger before–after studies of patients with any type of ICU-acquired infection (n = 201 and n = 1541, respectively), the conservative strategy was associated with significantly fewer patients given AT (notably active against anaerobes), a shorter median AT duration, and a greater number of in-ICU AT-free days [9, 10]. In both studies, the significantly lower all-cause mortality with the conservative strategy should be interpreted with caution, as disease severity may have differed between the two groups. A larger study specifically addressing VAP is therefore needed. Several methods exist for documenting antibiotic consumption. We considered length of treatment, defined as the number of days on AT regardless of the number of antibiotics or doses. Data accuracy might be improved by recording the days on each individual active antimicrobial agent. The mean defined daily dose [26] may be less satisfactory, as it is tedious to determine and may overestimate AT use in the ICU [27].

The numbers of days on broad-spectrum antimicrobials and on carbapenem did not differ significantly between our two groups. However, this result may be related to the bacterial ecology in our ICU. The incidence of infections due to MRBs is low in our region compared to several other parts of France [21]. The result is a lower overall use of AT in our ICU than in other similar centers in the country, as illustrated by the high median number of days alive without carbapenem in our study. Conceivably, the conservative strategy might provide greater AT sparing in regions where MRBs are more common than in ours.

Another factor that may have limited the ability of the conservative strategy to significantly decrease AT use in our study is the strong focus on antibiotic stewardship in our ICU. Our center participates actively in the REA-REZO network for monitoring and decreasing antibiotic resistance. Moreover, we have staff specifically trained in infectious diseases and AT use. Also, our data highlight the close attention given in our ICU to AT de-escalation whenever possible.

Few studies have directly assessed potential associations between AT timing and outcomes of patients with VAP. A pilot multicenter randomized trial included 186 surgical patients who had suspected VAP with no vasopressor requirement [5]. Day-30 mortality and ventilator-free days were not significantly different between the immediate and specimen-initiated strategies. In a large before–after study of surgical patients with any type of ICU-acquired infection, the conservative strategy was not associated with higher mortality or worsening of other patient outcomes compared to the immediate strategy [10]. Our results are consistent with those from recent studies but run counter to older, heterogeneous, observational data, often from small single-center studies [28–37], in which delayed AT, classified as inappropriate AT, seemed associated with higher mortality [38]. This apparent discrepancy may be related to variations in the definition of inappropriate AT, which included the conservative strategy, and to other methodological flaws such as population heterogeneity [32], failure to adjust for critical-illness severity at VAP diagnosis [35], unexpectedly high rates of inappropriate AT [36], respiratory sampling after AT initiation [34], and a variable incidence of VAP due to complex/multiresistant bacteria (of poor prognosis regardless of initial AT appropriateness). Finally, a small prospective study of AT timing in VAP found that the most common reason for delayed active AT was delayed prescription, which may have been a marker for suboptimal care [28].

The classical clinical and radiological indicators of VAP lack accuracy, and relying on them may therefore result in unnecessary AT [39, 40]. While underpowered, our study agrees with three others [5, 9, 10] suggesting that ICU-patient outcomes may not be worse with the conservative strategy, which may avoid unnecessary AT in over a quarter of patients [5]. Thus, although the available data do not warrant a change in practice, they suggest equipoise and an acceptable risk/benefit ratio of culture-initiated AT, thus supporting the ethical legitimacy of larger studies. Tailoring the AT strategy to disease severity is in line with the current interest in personalized sepsis management [39, 41–43]. It is worth noting that the dogma of starting AT within the hour in sepsis, suggested by non-ICU observational data from the Surviving Sepsis Campaign, has been softened in recent recommendations due to concern that it might result in AT overuse [12]. Other studies of sepsis also suggest that a reasonable delay between admission and AT initiation may reduce antibiotic misuse without impairing patient outcomes [44, 45], notably when vasoactive agents are not required [46]. In a randomized trial, prehospital AT initiation for sepsis did not improve survival, regardless of severity [47].

The limitations of our study include the mixed retrospective/prospective before–after design, as opposed to randomization. We are aware of a single pilot randomized trial comparing immediate and conservative AT [5]. Each participating ICU used both strategies according to a cross-over design. Compliance with both strategies was good, supporting the feasibility of a randomized trial focused on VAP. Second, our small sample size impaired our ability to detect statistically significant differences, notably for our primary outcome reflecting antibiotic sparing. That AT suitability was significantly better in the conservative group despite the low statistical power deserves note. Third, the recruitment in a single university-hospital ICU and low incidence of MRB infections in our region may limit the general applicability of our findings. The two strategies should be compared in ICUs where MRB infections are more common than in ours. Fourth, a 2-year period elapsed between data recording for the two groups. However, during this period, there were no changes in staffing levels, equipment, or ICU protocols that may have affected AT use. Moreover, most patients on iMV during the excluded period had COVID-19. Enrolling them would have biased our results, as COVID-19 is associated with greater severity [48], a higher incidence of VAP [17, 49], and a specific bacterial ecology [50]. Also, the excessive workload borne by ICU staff during the initial COVID-19 waves may have altered the quality of care [51]. COVID-19 was present in about a fifth of our patients in the conservative group (9/43, 20.93%), but the disease was less severe than in previous years, due to global vaccination effects [52]. Finally, the microbiological diagnosis in our study relied on distal respiratory sampling and culturing. Emerging rapid diagnostic tests such as multiplex polymerase chain reactions for nosocomial respiratory pathogens (e.g., Unyvero® and Biofire Film Array® Pneumonia *Plus* Panel) may provide the diagnosis within a few hours. However, no randomized controlled trials of these tests in ICU patients are available. Gram staining of respiratory samples may provide useful information at a low cost but has shown conflicting results about their predictive value and agreement with culture [22, 53]. Large randomized trials are needed to further evaluate the potential usefulness of Gram stains on respiratory samples from patients with suspected VAP.

## Conclusions

When VAP was suspected in patients having no severity criteria, a conservative strategy of waiting for culture results and starting AT only when these were positive was not associated with a larger number of AT-free days by day 28 compared to immediate AT. This result may be due to low statistical power. The conservative strategy was associated with a higher proportion of patients receiving suitable AT, defined as either AT active on recovered microorganisms, or no AT and negative culture results. Safety outcomes were not worse with the conservative strategy. However, given the low statistical power, conclusions on safety should await large randomized trials with interim analyses of safety outcomes.

### Supplementary Information

**Additional file 1: Table S1.** Organisms identified in the patients with positive distal respiratory sample results confirming ventilator-associated pneumonia (VAP). **Table S2.** Empirical antibiotic agents used. **Table S3.** Analysis of safety outcomes confined to patients with confirmed ventilator-associated pneumonia. **Table S4.** Adjusted analysis of antibiotic use and other outcomes

## Data Availability

Restrictions apply to the data that support the findings of this study, which were used under license for the current study and are, therefore, not publicly available. The data are, however, available upon reasonable request to the corresponding author (Maëlle MARTIN, maelle.martin@chu-nantes) provided ethics committee approval of the data sharing is obtained.

## Abbreviations

95%CI: 95% Confidence interval
ARDS: Acute respiratory distress syndrome
AST: Antibiotic susceptibility testing
AT: Antibiotic therapy
BAL: Bronchoalveolar lavage
CPE: Carbapenemase-producing *Enterobacterales*
iMV: Invasive mechanical ventilation
ESBL-e: Extended-spectrum beta-lactamase producing *Enterobacterales*
GISA: Glycopeptide-intermediate *S**taphylococcus aureus*
HR: Hazard ratio
ICU: Intensive care unit
IRAB: Imipenem-resistant *Acinetobacter **b**aumannii*
MRB: Multiresistant bacteria
MRSA: Methicillin-resistant *Staphylococcus aureus*
PaO_2_/FiO_2_: Partial pressure of oxygen in arterial blood/fraction of inspired oxygen
TPC: Telescoping plugged catheter
VAP: Ventilator-associated pneumonia
VRE: Vancomycin-resistant *Enterococcus*
