# Supplementary material for: Immediate vs. culture-initiated antibiotic therapy in suspected non-severe ventilator-associated pneumonia: a before–after study (DELAVAP)
Source: Ann Intensive Care. 2024 Feb 27;14:33. doi: 10.1186/s13613-024-01243-z (PMC10897643; doi:10.1186/s13613-024-01243-z)
Supplement: Supplementary file 1 — Additional file 1: Table S1. Organisms identified in the patients with positive distal respiratory sample results confirming ventilator-associated pneumonia (VAP). Table S2. Empirical antibiotic agents used. Table S3. Analysis of safety outcomes confined to patients with confirmed ventilator-associated pneumonia. Table S4. Adjusted analysis of antibiotic use and other outcomes [file 13613_2024_1243_MOESM1_ESM.docx]

**DELAVAP- SUPPLEMENTARY FILE 1**

**TABLE OF CONTENTS**

**Table S1: Organisms identified in the patients with positive distal respiratory sample results confirming ventilator-associated pneumonia (VAP)……………………………p 2**

**Table S2: Empirical antibiotic agents used……………………………………………...p 3**

**Table S3: Analysis of safety outcomes confined to patients with confirmed ventilator-associated pneumonia……………………………………………………………………..p 4**

**Table S4: Adjusted analysis of antibiotic use and other outcomes……………………..p 5**

**Table S1: Organisms identified in the patients with positive distal respiratory sample results confirming ventilator-associated pneumonia (VAP)**

| **Identified organism, n (%)** | **Immediate^a^**  **n=30** | **Conservative^b^**  **n=26** | **Total** |
| --- | --- | --- | --- |
|  |  |  |  |
| **Gram-positive bacteria**  *Staphylococcus* *aureus*  *Streptococcus* sp. | 8 (26.7)  5 (16.7) | 14 (56.0)  0 (0.0) | 22 (40.0)  5 (9.1) |
| **Gram-negative bacteria**  *Haemophilus influenza*  *Escherichia coli*  *Klebsiella pneumonia*  *Enterobacter* sp.  *Hafnia* sp.  *Serrati*a  Other | 8 (26.7)  6 (20.0)  2 (6.7)  4 (13.3)  1 (3.4)  -  3 (10.0) | 5 (20.0)  5 (20.0)  2 (8.0)  4 (16.0)  2 (8.0)  1 (4.0)  2 (8.0) | 13 (23.6)  11 (20.0)  4 (7.3)  8 (14.5)  3 (5.5)  1 (1.8)  5 (9.1) |
| **Non-fermenting bacteria**  *Pseudomonas* *aeruginosa* | 3 (10.0) | 1 (4.0) | 4 (7.3) |
| **Other** | 1 (3.3) | 2 (8.0) | 3 (5.4) |

^a^Antibiotic therapy was started immediately after the collection of distal respiratory samples.

^b^Of these 26 patients, 19 had antibiotic therapy started upon receipt of positive distal respiratory sample cultures and 7 had rescue antibiotic therapy started before receipt of the culture results, which were positive.

**Table S2: Empirical^a^ antibiotics used**

| **Antimicrobials given, n (%)** | **Immediate AT**  n=44 | **Conservative AT**  n=31 | **Total** |
| --- | --- | --- | --- |
|  |  |  |  |
| **First AT** |  |  |  |
| Piperacillin-Tazobactam | 24 (54.5) | 5 (16.1) | 29 (38.7) |
| Amoxicillin-clavulanate | 9 (20.4) | 12 (38.7) | 21 (28.0) |
| 3d generation cephalosporin | 7 (15.9) | 3 (9.7) | 10 (13.3) |
| 4th generation cephalosporin | 1 (2.3) | 4 (12.9) | 5 (6.7) |
| Carbapenem | 2 (4.5) | 4 (12.9) | 6 (8.0) |
| Others | 1 (2.3) | 3 (9.7) | 4 (5.3) |
|  |  |  |  |
| **Second AT** |  |  |  |
| None | 42 (95.4) | 25 (80.6) | 67 (89.3) |
| Aminoglycoside | 1 (2.3) | 3 (9.7) | 4 (5.3) |
| Fluoroquinolone | 0 (0) | 1 (3.2) | 1 (1.3) |
| Linezolid | 1 (2.3) | 0 (0) | 1 (1.3) |
| Other | 0 (0) | 2 (6.4) | 2 (2.6) |

AT: antibiotic therapy

^a^Empirical antibiotic therapy (AT) was defined as started before availability of the antibiotic susceptibility test results. Empirical AT was also started before the culture results in the immediate group (n=44). In the conservative group, empirical AT was started upon receipt of positive cultures in 19 patients, before receipt of positive cultures in 10 patients with worsening symptoms (rescue therapy), and before receipt of positive cultures in 2 patients in whom the protocol was not followed, i.e., in 31 patients in all.

**Table S3: Analysis of safety outcomes confined to patients with confirmed ventilator-associated pneumonia**

|  | **Immediate**  n=30 | **Conservative**  n=26 | *p* value |
| --- | --- | --- | --- |
|  |  |  |  |
| iMV duration (days), median [95%CI] | 10.0 [5.0–X] | 10.0 [6.0–23.0] | 0.61 |
| Ventilator free-days, median [IQR] | 17.50 [0.0–23.0] | 15.0 [0.0–22.0] | 0.98 |
| ICU stay, days, median [95%CI] | 14.0 [7.0–16.0] | 14.0 [7.0–21.0] | 0.66 |
| Day 28 mortality, n (%) | 6 (20.0) | 5 (19.2) | 0.71 |

iMV: invasive mechanical ventilation; 95%CI: 95% confidence interval; ICU: intensive care unit

**Table S4: Analysis of antibiotic use and other outcomes adjusted on PaO_2_/FiO_2_ at suspicion of ventilator-associated pneumonia (VAP), respiratory reason for intubation,**

**and time from intubation to VAP suspicion**

|  | **Immediate**  **n= 44** | **Conservative**  **n= 43** | ***p* value** |
| --- | --- | --- | --- |
|  |  |  |  |
| Days alive without AT by day 28, median [IQR] | 16.0 [0.0–20.0] | 18.0 [0.0–21.0] | 0.87 |
|  |  |  |  |
| Days alive without broad-spectrum AT by day 28, median [IQR] | 23.5 [5.0–26.0] | 25.0 [0.0–28.0] | 0.80 |
| Days alive without carbapenem by day 28, median [IQR] | 28.0 [9.0–28.0] | 28.0 [0.0–28.0] | 0.65 |
|  |  |  |  |
| iMV duration, days, median [95% confidence interval]  HR [95%CI] | 9.0 [6.0–24.0]  1.1 [0.7–1.9] | 9.0 [6.0–19.0] | 0.61 |
| Ventilator-free days, median [IQR] | 18.5 [0.0–23.0] | 16.0 [0.0–22.0] | 0.83 |
| ICU stay, days, median [95% confidence interval] | 9.0 [6.0–14.0] | 13.0 [8.0–17.0] | 0.72 |
| Day-28 mortality, n (%)  HR [95%CI] | 11 (25.0)  1.1 [0.4–2.8] | 11 (25.6) | 0.84 |

AT: antibiotic therapy; iMV: invasive mechanical ventilation; HR: hazard ratio; ICU: intensive care unit
